# Mediastinal Ectopic Pancreas with Abundant Endocrine Cells Coexisting with Mediastinal Cyst and Thymic Hyperplasia

**DOI:** 10.1155/2018/8270516

**Published:** 2018-05-27

**Authors:** Yosinta Snak, Ery Kus Dwianingsih, Auliya Suluk Brilliant Sumpono, Rovi Panji, Afif Rahman, Ahmad Ghozali, Samir S. Amr

**Affiliations:** ^1^Department of Anatomical Pathology, Faculty of Medicine, Universitas Gadjah Mada, Yogyakarta, Indonesia; ^2^Department of Radiology, Faculty of Medicine, Universitas Gadjah Mada, Yogyakarta, Indonesia; ^3^Department of Pathology and Laboratory Medicine, King Fahad Specialist Hospital, Dammam, Saudi Arabia

## Abstract

Mediastinal ectopic pancreas is a rare condition with only 28 cases reported in the literature. Here we report a 21-year-old female patient who presented with dyspnea and intermittent severe chest pain of 7 years' duration. Computerized tomography scan (CT-scan) of the chest revealed a mediastinal cyst. The cyst was resected and it demonstrated on histopathological examination the presence of pancreatic tissue with increased number of islets of Langerhans, coexistent with mediastinal cyst and thymic hyperplasia. We made a review of all previously reported cases of this entity.

## 1. Introduction

Ectopic pancreas is defined as pancreatic tissue which grows outside its normal location and without vascular or other anatomical connections to the pancreas. It had been also named in the literature as heterotopic, accessory, or aberrant pancreas [1]. It is a congenital anomaly found in approximately 2% of autopsies, and most (70–90%) are located within the gastrointestinal tract [2, 3]. Development of pancreas within the mediastinum is quite rare. To our knowledge, 28 cases have been reported in the literature, showing variable clinical, radiographic, and histopathological characteristic. Thymic hyperplasia is defined as an enlarged thymus composed of normal thymic lobules with preserved corticomedullary differentiation [2]. It may coexist with a mediastinal cyst [3]. Here we report a patient who presented with mediastinal ectopic pancreas with hyperplasia of islets of Langerhans and coexisted with mediastinal cyst and thymic hyperplasia.

## 2. Case Report

**Figure 1:**
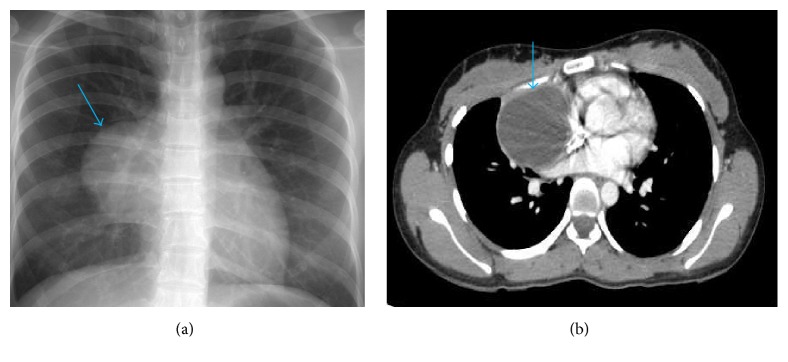
(a) Chest X-ray showed 6 × 4.5 cm white opaque lesion in mediastinal area (blue arrow) and (b) CT-scan revealed hypodense mass 6.7 × 7.5 cm, suggesting inferoanterior mediastinal cyst (blue arrow). Encapsulated cystic lesion compressed adjacent aorta and pericardium.

A 21-year-old female patient was admitted with chronic dyspnea and intermittent severe chest pain of 7 years' duration. Chest X-ray showed a mediastinal mass. Thoracic CT-scan revealed a cystic lesion, 6.7 × 7.5 cm located in inferior part of the anterior mediastinum (Figure 1). Laboratory workup including hematological, biochemical, and hormone profiles was all within normal limits. Surgical resection of this cystic mass was performed.

**Figure 2:**
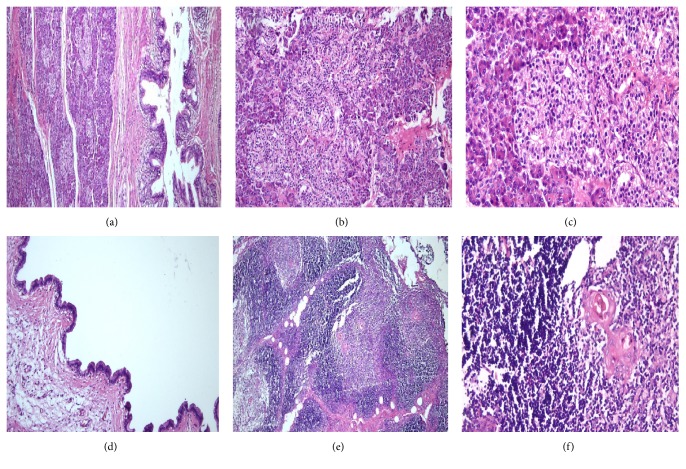
(a) An Island of pancreas tissue bordered by thick fibrous tissue with pancreatic interlobular duct around it (H&E-4x). (b) Pancreas tissue consists of exocrine gland with eosinophilic granular cytoplasm and endocrine cells (Islets of Langerhans) (H&E-10x). (c) Increase in the endocrine portion (Islets of Langerhans) of pancreatic tissue (H&E-40x). (d) Mediastinal cyst lined by simple cuboid and columnar epithelium (H&E-10x). (e) Hyperplasia of thymic gland was observed (HE-4x). (f) Normal appearance of lymphocytes and Hassall's corpuscles (H&E-10x).

**Figure 3:**
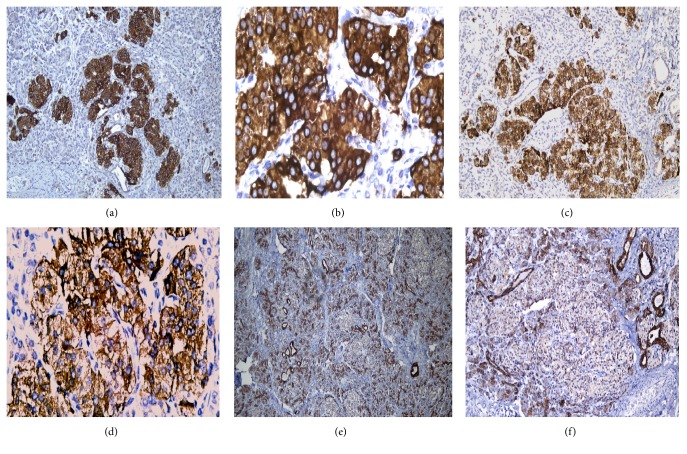
Immunohistochemical staining. (a and b) High expression of insulin in pancreatic beta cells of islets of Langerhans (10x–40x). (c and d) Strong positivity of chromogranin in islets of Langerhans (10x–40x). (e and f) Cytokeratin showed strong positivity in acinic cells and pancreatic duct; meanwhile endocrine cells showed very weak expression (4x–10x).

Gross pathological examination of the mass showed 2 fragments of opened soft cystic tissue, 6 × 5 cm and 4 × 3 cm in size. A thickened white to brown area within the wall of the cyst was noted. Histopathological examination revealed hyperplastic thymic tissue arranged into lobules, separated by fibrous tissue septa. The corticomedullary differentiation of thymus was well preserved, and Hassall's corpuscles were readily identified. Some empty and eosinophilic fluid filled spaces lined with cuboidal to columnar cells were identified. In the thickened area, pancreatic tissue, with both exocrine and endocrine elements, was identified. It consisted of acinar exocrine cells with interlobular ducts and abundant endocrine cells (Islets of Langerhans) (Figure 2). No features of fat necrosis were seen, and no cystic formations within the pancreatic tissue were noted. There were no teratomatous elements such as skin, neural tissue, bone, cartilage, and endodermal components. No malignancy was observed. Immunohistochemical staining for insulin showed strongly positive Beta cells of the islets of Langerhans within the ectopic pancreatic tissue (Figure 3). The patient had an uneventful postoperative course. Her last follow-up visit at six months showed normal chest CT- scan and no evidence of recurrence of the mass.

## 3. Discussion

Most cases of ectopic pancreas are located in the gastrointestinal tract, such as the stomach, duodenum, jejunum, and ileum [2]. However, it can be located in other sites, such as the gallbladder, esophagus, common bile duct, spleen, mesentery, and the mediastinum. The histogenesis of a mediastinal ectopic pancreas is currently unknown [4]. However, there are two main theories regarding the embryogenesis of this abnormal development. The first theory is the pluripotent epithelial cells of the ventral primary foregut that undergo anomalous differentiation, called heteroplasia, and this may lead to the formation of ectopic pancreatic tissue in the mediastinum. The second theory is that some cells from the pancreatic bud may migrate and locate at a different site [2].

There had been 28 cases of mediastinal ectopic pancreas reported in the world literature, demonstrating variable clinical, radiographic, and histopathological characteristic [2–25]. These cases are summarized in Table 1. It usually occurs in young adult, as in the current patient, with average age of 30 years, ranging from 5 to 66 years. There is slight female preponderance (58.6%) over males (41.4%), with a male to female ratio of 1 : 1.4. Clinical manifestations include chest pain, reported in 15 patients (51.7%), dyspnea in 8 patients (27.6%), and cough in 6 patients (20.7%). Other symptoms include pneumonia, hemoptysis, fever, throat discomfort, night sweat, shoulder pain, and neck swelling [5, 18, 23, 25]. Asymptomatic cases were also identified in 4 cases (13.4%) [3, 6, 13, 20].

Almost all patients with mediastinal ectopic pancreas showed unremarkable laboratory and physical examination. One case reported to have high blood pressure, fever, and leukocytosis [11]. Our patient showed common symptoms such as chest pain and dyspnea, but interestingly, she had these symptoms for the last 7 years. To our knowledge, this is the longest duration of the symptoms of this condition. Some patients had an opposite scenario with the disease manifesting itself for a short period of time of 2–4 weeks prior to the diagnosis [24, 25].

All reported cases of mediastinal ectopic pancreas were located in the anterior mediastinum. The size of the mass varied from 2 × 3 × 4 cm to 20 × 15 cm [7, 25]. More than 75% of the lesions were cystic, including 6 cases (27%) that had a solid component, as in this case. There were four cases that were solid without cystic changes. One case out of these four was reported to develop adenocarcinoma arising from the ectopic pancreatic tissues [18, 23, 24]. All benign cases were fully recovered after the operation and no recurrence was reported even years after surgery [20, 24]. The patient who developed adenocarcinoma died 15 months after surgery [16].

Radiologically, the cystic area and the solid portion of the lesions generally showed mild to moderate enhancement with contrast material. Cystic lesions in mediastinum may be clinically diagnosed as mediastinal abscess, benign teratomatous cyst, and true mediastinal cyst. Due to its common cystic nature, mediastinal ectopic pancreas currently can be suggested as one of the items in the differential diagnosis of mediastinal cystic lesions. Regarding the solid mediastinal ectopic pancreas, CT-scan cannot distinguish it from other solid masses such as thymoma, lymphoma, or malignant teratoma. In all these conditions, the diagnosis can be established only after surgery and histopathological examination [20].

Mediastinal cystic teratoma should be considered in the histological differential diagnosis of this lesion. Teratomas in general can contain pancreatic tissue in addition to other ectodermal, endodermal, and mesodermal elements. In one study of 469 teratomas from various sites, 17 cases contained pancreatic tissue, the majority of which (11 cases) were located in the anterior mediastinum [26]. Single case reports mediastinal teratomas with either increased exocrine pancreatic activity [27] or associated with aberrant islet differentiation or nesidioblastosis [28].

Our case has common cystic lesion as other mediastinal ectopic pancreas cases. Interestingly, the ectopic pancreatic tissue has a abundant component in its endocrine cells of the islets of Langerhans. These cells stained positively for insulin by immunohistochemical staining. The patient did not have a low glucose level and did not suffer from hypoglycemia. The adjacent thymus showed hyperplasia with well-preserved corticomedullary structures. This coexistence of thymic hyperplasia and ectopic pancreatic tissue had not been reported previously. We assume that, due to long and chronic course of the disease of 7 years, the thymus was induced to hyperplastic and reactive changes.

## 4. Conclusion

Mediastinal ectopic pancreas is extremely rare entity. Up to now, 28 cases have been reported, mostly occurring in young adults, and females are more prone to have the anomaly. Frequent clinical manifestation is chest pain and dyspnea. Benign cystic lesions are the most common feature of this entity. Mediastinal ectopic pancreas with increase in endocrine cells that coexisted with mediastinal cyst and thymic hyperplasia has never been reported previously.

## Figures and Tables

**Table 1:** Reported cases of mediastinal ectopic pancreas.

Number	References	Age	Sex	Clinical presentation	Lab/physical exam	tumor size (cm)	Gross
(1)	Shillitoe et al. 1957	15	Female	Dyspnea, night sweat	N/A	5.5	Cystic
(2)	Carr et al., 1977	56	Female	Asymptomatic	Normal	7 × 6.5	Cystic
(3)	Von Schweinitz et al., 1990	5	Male	Chronic pneumonia	N/A	5 × 5 × 5	N/A
(4)	Perez-Ordonez et al., 1996	16	Female	Asymptomatic	N/A	12	Cystic
(5)	Gong et al., 1997	26	Female	Chest pain, cough	N/A	20 × 15	N/A
(6)	Gong et al., 1997	26	Female	Chest pain	N/A	4.3 × 1.3	N/A
(7)	Wu et al., 1998	60	Female	Chest pain	N/A	10 × 5	Cystic
(8)	Cagirici et al., 2001	45	Female	Mild nonproductive cough, chest pain, headache	Normal	11 × 8	Cystic
(9)	Sentis et al., 2004	44	Male	Chest pain, dyspnea	N/A	10 × 8 × 7.5	Cystic
(10)	Tamura et al., 2005	39	Male	Chest pain	N/A	11 × 8	Cystic
(11)	Al-Salam et al., 2006	40	Male	High fever, neck swelling, dyspnea	high blood pressure, fever, leucoytosis	8 × 6 × 6	Cystic
(12)	Wang et al., 2007	17	Female	Chest pain, dyspnea	N/A	12 × 12 × 4	Cystic
(13)	Wang et al., 2007	24	Female	Chest pain, dyspnea	N/A	10 × 8 × 4	Cystic-solid
(14)	Chen et al., 2009.	32	Female	Asymptomatic	Normal	16 × 13 × 8 cm	Cystic-solid
(15)	Ehricht et al., 2009	25	Male	Lobar pneumonia	N/A	15	Cystic-solid
(16)	Fayoumi et al., 2010	51	Male	Chest pain, cough	N/A	10 × 7 × 5	Cystic
(17)	Fayoumi et al., 2010	42	Male	Shoulder pain	N/A	10 × 5 cm	Cystic-solid
(18)	Takemura et al., 2011	21	Female	Chest pain	N/A	3.5 × 3.5	Cystic
(19)	Byun et al., 2012	31	Female	Chest pain, productive mild cough	Normal	7 × 3 × 4	Cystic-solid
(20)	St Romain et al., 2012	66	Female	Chest pain	N/A	11 × 9	Solid
(21)	Szabados et al., 2012	32	Male	Pneumonia, hemoptysis and chest pain	N/A	4 × 4	Cystic
(22)	Rokach et al., 2013	22	Female	Asymptomatic, neck swelling	Normal	2.7 × 2.2 × 1.8	Cystic
(23)	Li et al., 2014	18	Male	Dyspnea	N/A	16 × 12 × 9	Cystic
(24)	Sibel et al., 2014	23	Male	Dyspnea	Normal	6 × 8	Cystic
(25)	Zhang et al., 2014	15	Male	Chest pain, coughing and fever throat	Normal	7 × 4.5	Solid
(26)	Zhang et al., 2014	16	Female	Discomfort, neck swelling	N/A	6	Solid
(27)	Koh et al.,	17	Male	Productive coughing	Normal	7.5 × 7 × 5.5	Solid
(28)	Wu et al., 2015	45	Female	Hemoptysis, lung infection	N/A	2 × 3 × 4	Cystic-solid
(29)	Present case	21	Female	Dyspnea and chest pain	Normal	6.7 × 7.5 cm	Cystic
